# Design of a Portable Low-Cost I-V Curve Tracer for On-Line and In Situ Inspection of PV Modules

**DOI:** 10.3390/mi15070896

**Published:** 2024-07-09

**Authors:** Monica De Riso, Mahmoud Dhimish, Pierluigi Guerriero, Santolo Daliento

**Affiliations:** 1 Department of Electrical Engineering and Information Technology, University of Naples Federico II, 80138 Napoli, Italy; monica.deriso@unina.it; 2 Laboratory of Photovoltaics, School of Physics, Engineering and Technology, University of York, York YO10 5DD, UK; pierluigi.guerriero@unina.it (P.G.); daliento@unina.it (S.D.)

**Keywords:** I-V curve tracing, PV module, monitoring and diagnosis, embedded system

## Abstract

Identifying underperforming photovoltaic (PV) modules is crucial to ensure optimal energy production and financial returns, as well as preventing potential safety hazards in case of severe damage. To this aim, current–voltage (I-V) curve tracing can be employed as in situ monitoring technique for the early detection of faults. In this paper, we introduce a novel low-cost, microcontroller-based I-V tracer for the diagnosis of individual PV modules. The tool features a unique power conditioning circuit, facilitating accurate data acquisition under static conditions as well as the even distribution of the measured points along the I-V curve. A specific active disconnecting circuit enables in situ and on-line measurement without interrupting the string power generation. The designed prototype is used to characterize a set of PV modules under real operating conditions. The measured I-V curves exhibit expected trends, with the measured data closely matching theoretical values and an estimated mean relative error less than 3%.

## 1. Introduction

Photovoltaic (PV) systems play a pivotal role in renewable energy generation, offering sustainable solutions for power generation across various applications. From residential rooftops to large-scale solar farms, PV modules are subjected to degradation and aging phenomena, impacting their performance and efficiency over time. Common drivers are the prolonged exposure to environmental stresses, such as temperature fluctuations [1], ultraviolet (UV) radiation [2] and atmospheric pollutants [3], and the intrinsic degradation mechanisms within PV materials, such as light-induced and potential-induced degradation, causing cell cracks and hotspot formation [4,5]. The estimated degradation rate is indeed found to vary between −0.48% and −0.88% per year, depending on the cell technology and the location of the PV system [6].

Since PV is a scalable technology, the PV modules are connected in series or in a parallel configuration to build a large power installation, with any fault among them negatively affecting the efficiency of the overall PV system. In other words, the PV system is only as efficient as its weakest component. To this aim, central to the efficient operation of PV systems is the ability to accurately characterize the performance of the individual PV modules. The literature reports several fault diagnosis methods, based on the analysis of the power actually produced using the maximum power point tracking algorithm (MPPT). As an example, Chaibi et al. [7] proposed a method based on the analysis of three predefined indicators extracted from the MPP coordinates to reveal the healthy or faulty operation of the PV system. Similarly, a sensor-less detection technique based on the current reduction between two MPPT sampling instants is proposed in [8] to detect catastrophic faults and shadings. However, these solutions are only sensitive to a limited numbers of faults and are only applicable at the string/inverter level, unless expensive micro-inverters are employed. The I-V curve, on the other hand, provides valuable insights into the electric characteristics of PV generators under varying environmental and operating conditions. This technique involves the user of a specific device called an I-V tracer. In general, an I-V tracer is made of a power conditioner, used to force the PV generator in a specific DC operating point, and a data acquisition system, to sense the resulting PV current and voltage and convert them into digital data. In the context of fault detection analysis, several I-V tracers are available in the market and in the scientific literature. Commercial devices are renowned for their reliability thanks to their adherence to industrial standards and the measurement accuracy ensured by the manufacturer. However, since most of them are designed for PV systems with large voltage and current capacities, they are expensive, thus limiting accessibility for some users.

To address this challenge, custom-designed I-V tracers based on low-cost general purpose electronic boards have been proposed. For example, Gonzalez et al. [9] presented an Internet of Things (IoT) open-source hardware and software I-V tracer for remote and real-time monitoring without requiring operator intervention. The major drawback of such an approach is the lack of an embedded power conditioner, thus relying on an expensive and bulky electronic load. An IoT-based solar monitoring system to remotely perform and communicate the I-V curve of PV modules is also presented in [10]. In this case, the power conditioner is based on a capacitive load switching between two different PV modules, thus requiring the interruption of energy production for both the tested modules. The I-V tracer presented in [11] is based on two sets of capacitor load and a sampling resistor. The proposed approach is demonstrated to provide high measurement accuracy in case large capacitance value is employed. However, the inability to reach a zero-load resistance prevents the acquisition of the short circuit current. Similarly, the I-V tracer presented in [12] exploits two sets of capacitors to also measure the second and fourth quadrant of the I-V curve by exploiting the charge transfer between the two capacitors. However, this strategy has been validated only on small solar cells and not for monitoring purposes. The capacitive approach is also adopted by Caceres at al. in [13] and Sayyad et al. in [14] for measuring PV modules with power up to 520Wp. Since the capacitive load stores the energy produced by the PV module during the measurement, a massive discharging resistive net is employed, thus leading to a lack of portability. Other examples of I-V tracers based on capacitive load can be found in [15] and [16]. By contrast, the device presented in [17] embeds a MOSFET transistor working with pulse-width modulation (PWM) as a power conditioner, as well as control relays to disconnect the PV module from the string during the I-V curve. Despite the low sweeping speed, namely 200 ms, the points measured by the device are randomly distributed along the I-V curve with a likely loss of information. The same drawback can be found in [18], where the device is implemented as a set of MOSFET transistors along different load resistors and in [19], where a single power MOSFET is employed, thus resulting in a lack of measured points on the horizontal branch of the I-V curve. This challenge is addressed by Vega et al. [20], where a proper feedback loop is employed to evenly distribute the measurement points along the I-V curve. A different approach is used in [21], where the power conditioner is based on a dc-dc converter. The proposed topology consists of varying the voltage on the PV terminals by controlling the duty cycle applied to the dc-dc converter. Its high dynamics guarantee a fast sweeping time, but the power switching leads to an inherent ripple in the PV voltage and the current degrading the accuracy. It is worth noting that the major disadvantage of the above-mentioned devices is the inability to perform an on-line measurement, thus resulting in the shutdown of the PV plant and string disassembly. Shutdown means a loss of power generation and a minimization of the return on investment (ROI).

In this paper, an innovative low-cost, microcontroller-based I-V tracer for the monitoring of an individual PV module is presented. The novelty of the proposed solution lies on the capability of addressing all the aforesaid drawbacks, which are summarized in the following aspects: (i) all the solutions reported in the literature require the shutdown and disassembly of the string to which the tested PV module is connected; (ii) they randomly distribute the measured points along the I-V curve, thus reducing the detectability of faults and bypass activation events; (iii) and they are affected by low measurement accuracy when switching topology is employed, and do not guarantee the static acquisitions of the measured points (as in the case of capacitive approach). To overcome these challenges, the proposed I-V tracer is based on a double-leg linear circuit employing Darlington transistors and resistive load, allowing for the acquisition of the operating points in static conditions [22]. The tool is suitable to be employed for in situ measurement, thanks to a battery-powered circuit and efficient wireless communication. The device embeds a novel active disconnecting circuit composed of a power MOSFET transistor, and a bypass diode to bypass the string current during the I-V curve measurement. Additionally, a proposed control algorithm is implemented to evenly distribute the measured points along the I-V curve, thus guaranteeing a sweeping time less than 1 s. The developed I-V tracer is tested and validated on a set of PV modules concerning different technologies under different levels of irradiance and environmental conditions. 

The structure of the paper is as follows: Section 2 describes the materials and methods, focusing on the principle of the workings of the proposed power conditioner and its implementation in the device. This section is also devoted to the description of the experimental setup. Section 3 presents the designed prototype as well as the obtained experimental I-V curves. Section 4 is devoted to the discussion of the obtained results, and finally the conclusions are drawn in Section 5. 

## 2. Materials and Methods

### 2.1. Principle of Operation

**Figure 1:**
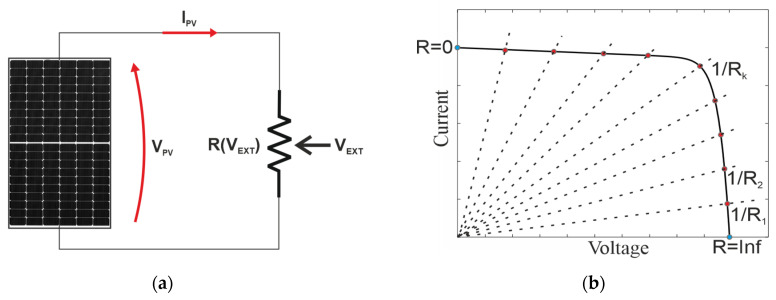
Principle of operation of an I-V curve tracer. (**a**) Variable load controlled by means of external parameter (*V_EXT_*); (**b**) sweeping of the operating points (in red) along the characteristics given as the intersection between the I-V curve of the PV generator and the load curves (black dashed lines) obtained at different R_k_ values. The open circuit and short circuit points (in light blue) are obtained for R = ∞ and R = 0, respectively.

The behavior of a PV generator under specific irradiance and temperature conditions is mainly described by its I-V characteristic. The I-V characteristic provides the static relationship between the generated current and the voltage across the PV generator, as well as indicating the maximum output power. A simple PV generator, such as a single solar cell, can be modelled according to the single diode model [23], described by the following equation:

(1) $$I=I_{PH}-I_{0}\left(e^{\frac{V+R_{S}I}{nV_{T}}}-1\right)-\frac{V+R_{S}I}{R_{SH}}$$

where *I* and *V* are, respectively, the output current and the voltage across the PV cell, *I_PH_* is the photo-generated current, *R_S_* is the series resistance accounting for the voltage drop across the transport resistance of the solar cell, *R_SH_* is the shunt resistance representing the effect of leakage current in the p-n interface [24], *n* is the ideality factor of the diode, *V_T_* is the thermal voltage and *I*_0_ is the saturation current. The I-V curve can be traced, connecting a variable load to the terminals of the PV generator, as schematized in Figure 1a. The operating point is given as the intersection between the I-V curve and the load curve (*I* = *V*/*R*), as graphically depicted in Figure 1b. Assuming that R is controlled by means of an external variable (*V_EXT_*), it is possible to trace the I-V characteristic from the short circuit current to the open circuit voltage by varying the value of R, ideally from 0 to infinity.

**Figure 2:**
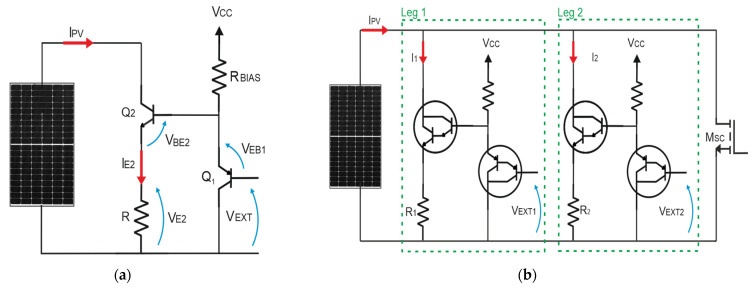
Circuit diagram of the developed I-V curve tracer. (**a**) Basic schematic of the variable load; (**b**) two legs variable load with additional branch for the short circuit measurement.

In our I-V curve tracer, the variable load is implemented, as in Figure 2. 

The circuit is composed of a pair of BJT transistors (Q_1_ is PNP and Q_2_ is NPN, Figure 2a), a resistive load R and a bias resistor R_BIAS_. The external variable *V_EXT_*, as shown in Figure 2b, used to control the load, is an analogue voltage applied to the base-collector of Q_1_. 

Applying the Kirchhoff’s law of voltage, the following equation is inferred:

(2) $$V_{E2}=V_{EXT}+V_{EB1}-V_{BE2}$$

Assuming that Q_1_ and Q_2_ are identical and working in the forward active region, *V_EB_*_1_ can be considered equal to *V_BE_*_2_. Therefore, the external voltage *V_EXT_* is virtually applied across the load resistor *R*. Additionally, since the forward current gain of Q_2_ is close to unity, *I_E_*_2_ can be considered equal to *I_PV_*. From Equation (3), it can be inferred that the PV current is linearly controlled by *V_EXT_*.

(3) $$I_{PV}=\frac{V_{EXT}}{R}$$

To trace the I-V characteristics, *V_EXT_* is initially set to 0 to measure the open circuit voltage. Afterward, *V_EXT_* is increased in increments until the PV current gets saturated at its short circuit value (*I_SC_*). From Equation (3), it can be deduced that the sensitivity of the I-V curve tracer, expressed as $\partial I_{PV}/\partial V_{EXT}$, does depend on R. The typical I-V curve of a PV generator, presented in Figure 1b, exhibits two distinct regions, one characterized by a steep increase in the current with minimal voltage variation (the vertical branch from the open circuit point to the MPP) and one presenting a gradual increase in the current with large voltage variation (the horizontal branch from the MPP to the short circuit condition). Consequently, I-V curve tracing could result in an uneven distribution of the points along the characteristics, mainly concentrated in the vertical branch, and only a few points would be captured in the horizontal one. 

The circuit proposed in Figure 2 can be extended by adding an additional parallel leg identical to that depicted in the figure, but with a smaller resistive load, to further increase the sensitivity of the instrument. The whole circuit, depicted in Figure 2b, comprises two parallel resistive loads with $R_{2}=10R_{1}$ independently controlled by two external voltages (*V_EXT_*_1_ and *V_EXT_*_2_). Since Leg 2 provides a smaller current resolution compared to Leg 1 because $∆I_{2}=∆I_{1}/10$, Leg 1 can be exploited to capture the points in the vertical region of the I-V curve, whereas Leg 2 can be used to capture the points in the horizontal one. Moreover, since the power generated by the PV generator during the I-V tracing is fully dissipated in the circuit, the additional leg allows us to split the power dissipated by the transistors, thus mitigating the thermal stress. Additionally, the BJT are replaced with Darlington transistors to provide higher impedance and higher forward current gain. 

It must be pointed out that as soon as the operating point approaches *I_SC_*, the Darlington transistors enter the saturation region. For this reason, the proposed circuit is not able to impose the short circuit condition, because the minimum measured voltage across the PV generator is $V_{PV,min}=RI_{SC}+V_{CE,sat}$. To tackle this issue, the variable load proposed in Figure 2 is provided with an additional leg made of a power MOSFET with small ON resistance (in the range of few mΩ), thus providing a voltage, i.e., $V_{PV,min}=R_{ON}I_{SC}$, closer to the short circuit condition.

### 2.2. Device Implementation

**Figure 3:**
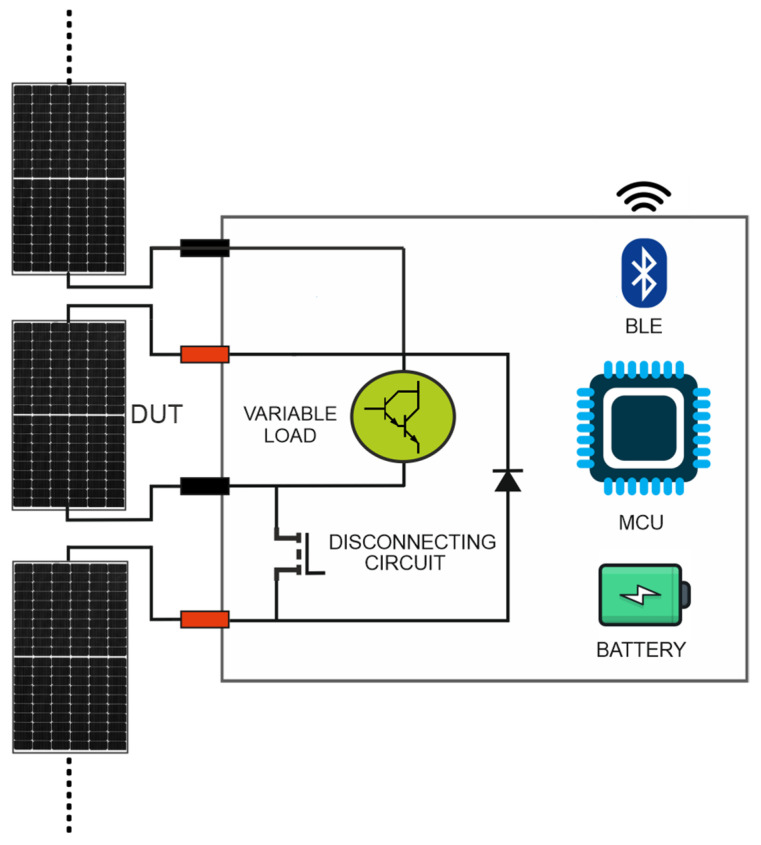
Schematic diagram of our developed in-house I-V curve tracer.

The variable load proposed in the previous subsection represents the hardware conditioner of our I-V tracer. The complete schematic diagram of our instrument and how it is connected to the PV generator under test is depicted in Figure 3. As can be seen, it is also provided with a disconnecting circuit, a control unit, a wireless communication module and a power supply section. 

#### 2.2.1. Disconnecting Circuit

**Figure 4:**
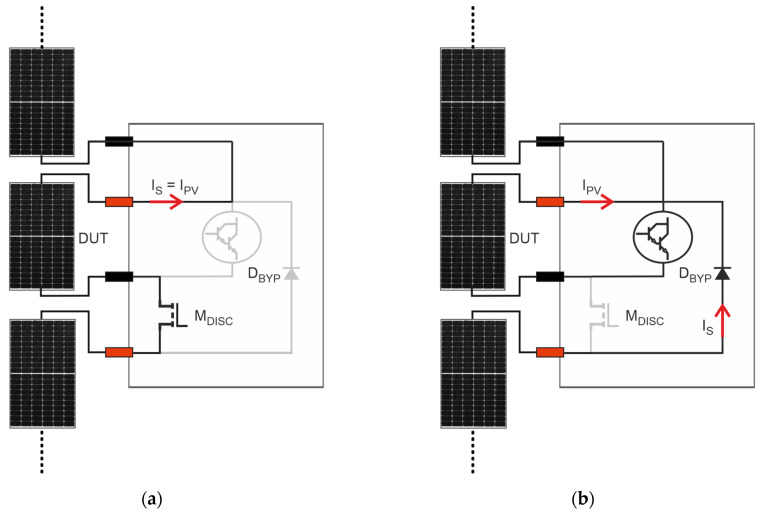
Principle of working of the disconnecting circuit. The grey color indicates the inactive components and the branches where no current is flowing. (**a**) Normal operation of the PV module embedded into the string, i.e., the disconnecting circuit is inactive; (**b**) during the measurement phase, the disconnecting circuit is active.

A traditional I-V curve tracer requires that the PV module under test is physically disconnected from the PV string, thus implying the shutdown of the PV system and a long and costly string disassembly. On the contrary, our instrument allows us to perform in situ and on-line I-V curve tracing thanks to a specific disconnecting circuit, preventing the interruption of the energy production of the PV string where the PV module under test is embedded. The disconnecting circuit is composed of a power MOSFET M_DISC_ and a bypass diode D_BYPASS_. The principle of operation is illustrated in Figure 4. 

During the normal operation (Figure 4a), M_DISC_ is ON, guaranteeing that the PV module under test is electrically connected to the string. In this phase, M_DISC_ acts as an almost ideal short circuit, because its ON resistance is in the order of few mΩ and both D_BYP_ and the variable load are inactive, guaranteeing that *I_PV_* is equal to the string current I_S_. At the start of the measurement, the control unit turns OFF M_DISC_ to electrically isolate the PV module, thus forcing the activation of D_BYPASS_ (Figure 4b). The bypass diode prevents the interruption of the I_S_ by creating an unimpeded path around the isolated PV module. During this period, the string keeps producing power, which is only reduced by the bypassed module’s contribution. Since the measurement takes less than 1 s, the operating point of the string, usually handled by the inverter, is immediately recovered.

#### 2.2.2. Control Unit

The control unit is based on a microcontroller (MCU) in charge of handling the measurement process, summarized as follows:

1. The START command is sent to the MCU from the remote controller.
2. The MCU electrically disconnects the PV module under test by deactivating the disconnecting MOSFET (M_DISC_).
3. A preliminary measurement of *V_OC_* (*V_EXT_*_1_ and *V_EXT_*_2_ are set to 0 and M_SC_ is deactivated) and *I_SC_* (*V_EXT_*_1_ = *V_EXT_*_2_ = 0 and M_SC_ is activated).
4. The MCU controller computes the current and voltage resolution as follows:
  (4) $${∆I}_{PV}=\frac{{∆I}_{SC}}{N/2}\;{∆V}_{PV}=\frac{{∆V}_{OC}}{N/2}$$
  where *N* is the number of points (set to 128). ∆*I_PV_* corresponds to the minimum current distance between two consecutive operating points captured in the vertical branch of the I-V curve, and ∆*V_PV_* corresponds to the minimum voltage distance between two consecutive operating points captured in the horizontal branch of the I-V curve. Equation 4 assumes a square-shaped I-V characteristic.
5. The MCU calculates the required voltage steps:
  (5) $${∆V}_{EXT1}={∆I}_{PV}R_{1}\;∆V_{EXT2}=∆V_{PV}$$
6. The complete flowchart of the I-V sweeping is systematically described in Algorithm 1. The first data point inserted into the buffer, *k* = 0, is the *V_OC_* previously acquired. The MCU measures the second data point (*k* = 1) incrementing *V_EXT_*_1_ and imposing *V_EXT_*_2_ = 0, assuming that it lies on the vertical branch of the I-V curve. The next task is the identification of the I-V branch. The MCU calculates the slope at the actual operating points as follows:
  (6) $$slope=\frac{I_{k-1}-I_{k}}{V_{k}-V_{k-1}}$$
  where (*V_k_*_−1_; *I_k_*_−1_) is the data point acquired at the *k* − 1st iteration, whereas (*V_k_*; *I_k_*) is the data point acquired at the *k*-th iteration. The slope is compared to a threshold value, corresponding to the knee of the I-V curve, calculated as in [25]:
  (7) $$knee=\frac{I_{SC}}{V_{OC}}$$

If *slope* > *knee*, the operating point lies on the vertical branch and the MCU increments *V_EXT_*_1_ (see line 15 in Algorithm 1). The measured data point is stored in the buffer if the distance in current from the previous acquired data is equal or greater than $∆I_{PV}$. By contrast, if *slope* < *knee*, the operating point lies on the horizontal branch and the MCU increments *V_EXT_*_2_ (see line 13 in Algorithm 1). The measured data point is stored in the buffer if the distance in current from the previous acquired data is equal or greater than $∆V_{PV}$. If not, the point is discarded, and the measurement is repeated.

7. Once the measurement is completed, the MCU turns ON M_DISC_ to restore the normal operation of the PV module under test.
8. The data points are sent to the remote controller over the wireless link.

The MCU generates the analogue voltages, *V_EXT_*_1_ and *V_EXT_*_2_, by means of a 12-bit double-channel digital to analogue converter (DAC) and measures the data points (voltage and current) through two independent 12-bit analogue to digital converter (ADC) channels working at 500 kS/s. 

**Algorithm 1.** Flowchart of the I-V sweeping.Pseudo-code of the Algorithm1*k* = 12*V_EXT_*_1_ = 03*V_EXT_*_2_ = 04*V* [0] = *V_OC_*5*I* [0] = 06WHILE *k* < *N*
7    IF *k* == 18        *V_EXT_*_1_+ = ∆*V_EXT_*_1_9        *V_EXT_*_2_ = 010    ELSE11     COMPUTE $slope=\frac{I\left[k-1\right]-I[k]}{V\left[k\right]-V[k-1]}$
12        IF *slope* < *knee*
13            *V_EXT_*_2_+ = ∆*V_EXT_*_2_14            ELSE15                *V_EXT_*_1_+ = ∆*V_EXT_*_1_16        END IF-ELSE17    END IF-ELSE18SEND *V_EXT_*_1_ AND *V_EXT_*_2_ TO DAC19RECEIVE *V_NEW_* AND *I_NEW_* FROM ADC20COMPUTE $∆I_{DIFF}={I\left[k-1\right]-I}_{NEW}$
21COMPUTE $∆V_{DIFF}=V_{NEW}-V\left[k-1\right]$
22    IF $∆I_{DIFF}\geq ∆I_{PV}$ OR $∆V_{DIFF}\geq ∆V_{PV}$
23        *V*[*k*] = *V_NEW_*24        *I*[*k*] = *I_NEW_*25        *k*++26    END IF27END WHILE

#### 2.2.3. Wireless Communication Module

**Figure 5:**
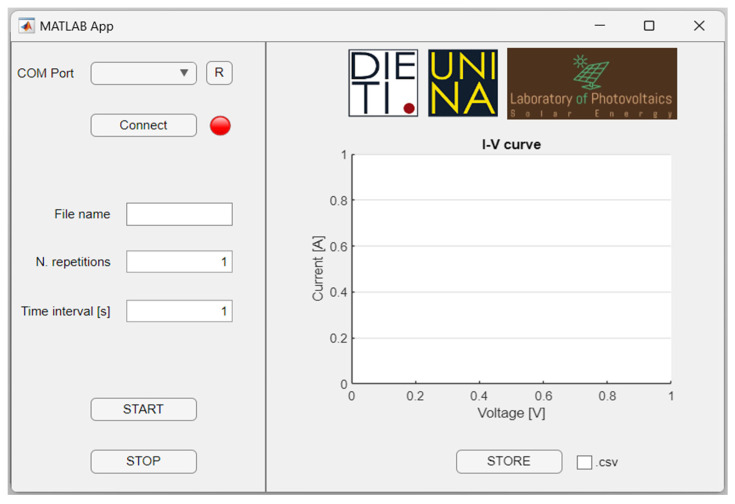
MATLAB GUI for the developed I-V curve tracer.

The wireless communication is implemented through a low-power Bluetooth (BT) protocol, enabling long distance communication of up to 1 km [26]. This module allows our I-V tracer to communicate with a remote controller through an ad hoc user-friendly graphic user interface (GUI) implemented in MATLAB R2022a (Figure 5). The BT communication is established as serial port, with a data buffer size of 1024 byte per acquisition. From the GUI, the user can establish the BT communication with our instrument, set the measurement parameters, start the measurement, visualize the data in real-time and store the data either in MATLAB or csv format for offline processing. Additionally, the user can set the number of acquisitions for repetitive measurements and the time interval between two consecutive acquisitions.

### 2.3. Experimental Setup

**Figure 6:**
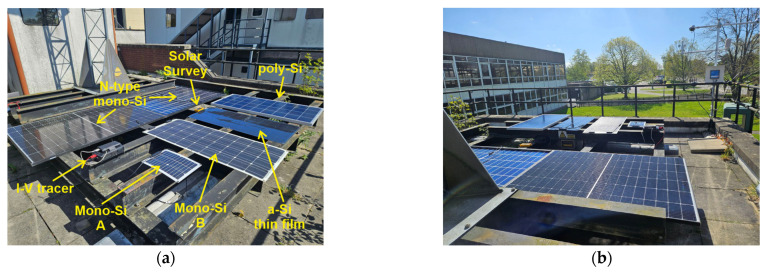
Experimental setup. (**a**) Tested PV panels; (**b**) outdoor experimental setup layout.

The efficacy of the developed I-V tracer is proven through an extensive experimental campaign carried out at the Laboratory of Photovoltaics at the School of Physics and Technology at the University of York, York (UK). The I-V tracer is used to test six PV modules of distinct technologies in outdoor conditions under three levels of irradiance, namely 700 W/m^2^, 500 W/m^2^ and 300 W/m^2^. Since the PV efficiency is significantly affected by the level of irradiance [27,28], these values are selected to provide a comprehensive understanding of the modules’ performance under high, medium and low illumination conditions, respectively. The set of PV panels comprises two free-standing bifacial N-type mono-Si PV modules, two flexible mono-Si PV modules of different power ratings, one flexible a-Si thin film PV module and one rigid poly-Si PV module, as labelled in Figure 6a. The ratings of the modules are reported in Table 1.

The measurement is carried out on the rooftop of the Laboratory of Photovoltaics in York, UK, (latitude and longitude coordinates: 53.95° and −1.08°) during a day in April in clear sky conditions. The environmental parameters, such as the incident irradiance, the ambient temperature and the module temperature, are recorded by means of Solar Survey 200R. The tested modules are conveniently oriented towards the south and slightly tilted, with an inclination angle of 4°, as shown in Figure 6b, to facilitate the measurement process.

It is worth noting that the irradiance is sensed by Solar Survey 200R by means of a c-Si reference cell calibrated under the standard solar spectrum AM1.5G. Spectral mismatch issues may arise when performing outdoor measurement because the solar spectrum may not be the same as that of the reference spectrum (AM1.5G). In this case, the international standard IEC 60904 [29] suggests estimating the spectral mismatch factor to correct the measured solar spectrum, as discussed in [30]. The correction procedure requires knowledge of the spectral response of the tested PV module, usually measured experimentally using a spectrometer. Due to the unavailability of this information and the limitation of our lab equipment, it has been assumed that the solar spectrum impinging the tested PV modules is AM1.5, because the measurements were performed in clear sky conditions with a solar zenith angle spanning from 47° to 48° at our geographical coordinates at the time of our experiment (April), closely matching the angle at which the standard spectrum AM1.5 is obtained (48.2°). Hence, any effect of spectral mismatch is neglected.

## 3. Results

### 3.1. Prototype Design

**Figure 7:**
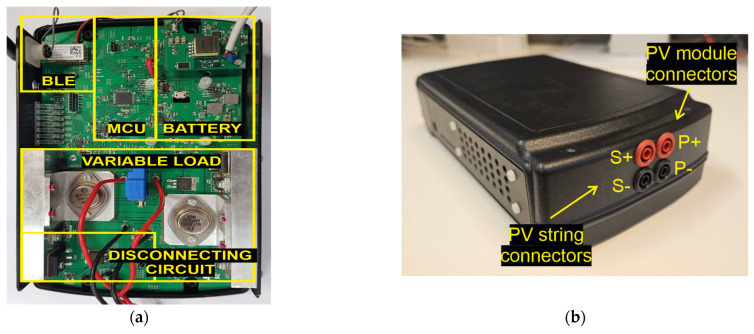
Physical layout of the developed I-V curve tracer. (**a**) The implemented prototype; (**b**) IP67-rated enclosure and connectors to PV module and PV string.

The prototype of the proposed I-V curve tracer has been realized on printed circuit board (PCB), shown in Figure 7a, embedded in a small plastic enclosure rated IP67, depicted in Figure 7b. The MCU employed in the prototype is 70 MHz clock-speed dsPIC33EP by MICROCHIP, supporting two independent 12-bit ADC modules used for the simultaneous acquisition of the PV current and voltage. The circuit can measure PV voltage up to 40 V employing a specific resistive voltage divider, whereas the PV current is sensed by means of a Hall-effect sensor IC placed in series with the current path of the PV module. The Hall-effect current sensor has been selected among other current sensing topologies, such as calibrated shunt resistors, because it is suitable for a wide current range (the maximum sensed current is 10 A) without introducing significant power loss, and it benefits from galvanic isolation, thus preventing signal noise as well as ground loop feedback. The data acquired by the ADC channels are digitally filtered to remove the noise and partially processed on-chip to assure that they are acquired in static conditions. The prototype is battery-powered by a 3.7 V 2050 mAh Li-Ion battery. The main components used in the prototype are listed in Table 2. As highlighted in Table 3, the total cost of the board is approximately EUR 355, and it is expected to diminish in the case of mass production. Table 3 clearly demonstrates the cost-effectiveness of the proposed board with respect to commercial devices, which typically rely on bulky and expensive load capacitors. All the prices are up to date as of the time of publication of this work.

Although the prototype was designed to measure PV modules with *V_OC_* and *I_SC_* up to 40 V and 10 A, respectively, the proposed circuit solution can easily be extended to larger systems, such as PV strings, by replacing transistors with higher power ratings and selecting current and voltage sensors with the appropriate measurement range.

### 3.2. Measured I-V Curves

To validate our I-V tracer, the I-V curves of PV modules of distinct technologies have been obtained under real operating conditions. Since the measurements have been carried out in outdoor conditions and in different periods of the day, the level of irradiance varies slightly for each PV module and they can be grouped in three nominal values, namely 300 W/m^2^, 500 W/m^2^ and 700 W/m^2^. For each curve, three main electric parameters are extracted (*V_OC_*, *I_SC_* and P_MAX_), and the PV temperature as well as the environmental parameters are reported in Table 4. G_f_ indicates the irradiance received on the front side of the PV modules, whereas G_b_ represents the irradiance received on the back side of the bifacial PV modules.

**Figure 8:**
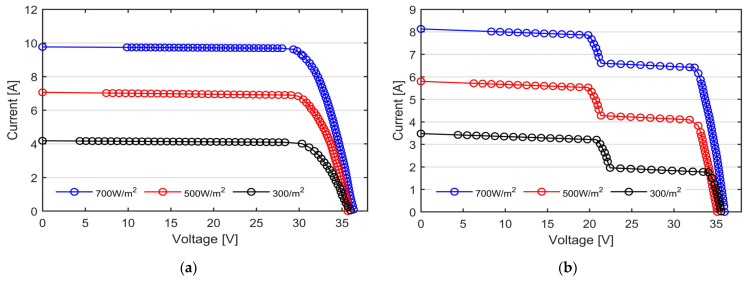
I-V curves of the N-type mono-Si bifacial PV modules under three nominal irradiance levels, namely 700 W/m^2^ in blue, 500 W/m^2^ in red and 300 W/m^2^ in black, measured with our custom-made I-V tracers. (**a**) Module #1 with the front side exposed to the sun; (**b**) module #2 with the back side exposed to the sun. The ladder-shape curves are caused by the wires partially shadowing the exposed surface.

Figure 8 reports the I-V curve measured on the bifacial PV modules, obtained by irradiating each side independently. To better appreciate the capability of our I-V tracer to evenly distribute the measured points along the characteristics, the actual measured points are marked by circular points. It is worth noting that all the experimental curves reported in this section are directly obtained from the I-V tracer and no curve approximation is performed [31]. As previously mentioned, the data are processed solely by the microcontroller unit at the time of measurement, utilizing an ad hoc digital average filter applied to 16 consecutive samples for each data point. This approach ensures that the data are acquired under static conditions and enhances the signal-to-noise ratio.

At the same levels of irradiance, module #2 shows a reduction of almost 16% in the short circuit current due to the worse optical properties of the rear side compared to the front. The tested bifacial panels show, in fact, a bifaciality factor of 0.8, as reported by the manufacturer [32]. Additionally, the I-V curves of module #2 show the typical ladder-shape characteristic, due to the presence of the wires partially shadowing the exposed surface (the junction box is placed in the back side). As can be observed from Figure 8 as well as from Table 4, the *V_OC_* decreases whilst the level of irradiance decreases, except for the black curve. It is well known that the *V_OC_* has a strong negative dependence on the temperature and only a fair positive dependence on the solar irradiation. Consequently, the black curve shows a higher *V_OC_* compared to the red one due to the lower PV temperature (28 °C and 35 °C, respectively, as reported in Table 4).

**Figure 9:**
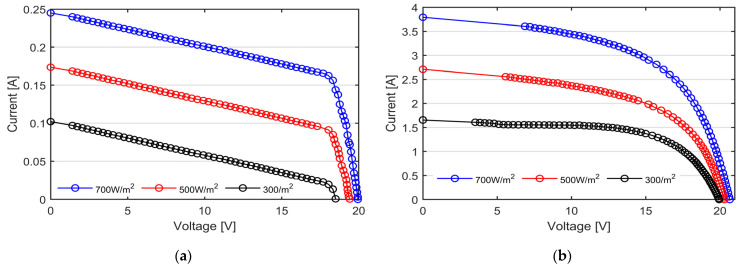
I-V curves of the mono-Si modules under three nominal irradiance levels, namely 700 W/m^2^ in blue, 500 W/m^2^ in red and 300 W/m^2^ in black, measured with our custom-made I-V tracers. (**a**) Module #1 with rated power 20 Wp; (**b**) module #2 with rated power 100 Wp.

The curves reported in Figure 9a,b are those acquired from the flexible mono-facial mono-Si PV modules rated, respectively, 20 Wp (indicated as Mono-Si B in Figure 6a) and 100 Wp (Mono-Si A in Figure 6a).

By comparing the measured values with those reported from Table 2, it is clear that both the PV modules are affected by a significant power loss. The shape of the measured I-V curve differ from the one of a healthy module. For example, in module #2, the extracted values of the saturation current (I_0_ = 0.3 mA) and shunt resistance (R_SH_ = 30 Ω) indicate a reduction in the charge carriers’ extraction at the contacts of the silicon cells, as well as a significant amount of leakage current through the shunted paths across the PV cells.

**Figure 10:**
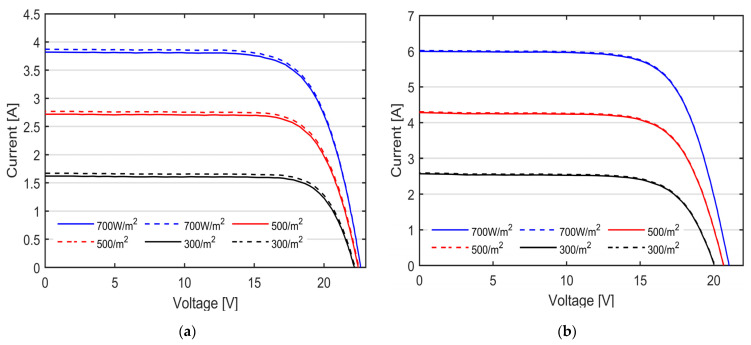
I-V curves under three nominal irradiance levels, namely 700 W/m^2^ in blue, 500 W/m^2^ in red and 300 W/m^2^ in black, measured with our custom-made I-V tracers (solid lines) compared to the theoretical I-V curve in the same operating conditions (dashed lines). (**a**) a-Si thin film PV module; (**b**) Poly-Si PV module.

The I-V curves obtained from the remaining tested technologies, namely a-Si thin film and poly-Si, are reported, respectively, in Figure 10a,b. The measured I-V curves are also compared to the theoretical I-V curves (reported in dashed line), obtained from the datasheet of the PV modules, employing the single diode model (see Equation (1)) under matching solar irradiance and temperature conditions. The measured irradiance and PV temperature are reported in Table 4. The single diode model is selected for its ease of implementation and lower computational resource requirements, while still providing sufficient accuracy and consistent results for the electrical modelling of the tested modules.

It is well-known that a-Si technology experiences an initial degradation caused by light soaking, i.e., the Staebler–Wronski effect, which reduces the power until it reaches a stabilized level, typically after few hundred hours of illumination [33]. The degraded performance might be partially recovered from the effects of thermal annealing, such as during summertime after a long exposure to weather conditions [34]. To address the Staebler–Wronski effect, the IEC 61646 standard [35] requires that the electrical parameters provided by the manufacturer refer to the stabilized conditions. Since the sunlight exposure of the tested PV module was not permanent but limited to the time of the experiments, a good agreement between the nominal and testing conditions is assumed.

As can be observed in Figure 10, the curves show the same trend with very scarce differences, in particular in the horizontal flat of the I-V curves. To better appreciate the discrepancy between the theoretical data and the ones measured with our I-V tracer, the mean value and the standard deviation of the relative percentage error is computed for each tested PV module. The results are reported in Table 5.

The mean relative error is an indication of the accuracy of our instruments, exhibiting a maximum discrepancy of approximately 3% in the mono-Si technology. By contrast, the best performance is shown in the Poly-Si technology, showing a good match between the measure data and the theoretic ones.

## 4. Discussion

The experimental I-V curves, presented in the previous section, assess the suitability of the proposed I-V tracer to perform the in situ and real-time characterization of PV modules of distinct technologies under different environmental conditions. As shown in Figure 8 and Figure 9, the proposed I-V tracer has the capability of distributing the measurement points along the I-V curves both in uniform and partial shading conditions, thus allowing for an accurate estimation of the module’s parameters, such as the series and shunt resistances, as well as the proper identification of the bypass activation events. The lack of data near the short circuit points does not lead to loss of information because of the linear trend of the I-V curve near *I_SC_*. The utilization of a 70 MHz clock-speed MCU, along with two independent 12-bit ADC, enables the simultaneous acquisition of PV current and voltage. The data are digitally filtered during the acquisition thanks to a real-time moving average filter comprising 16 consecutive points, thus enhancing the signal-to-noise ratio (SNR) and avoiding post-processing delays. Consequently, our I-V tracer measures the I-V curve in less than 1 s, with high precision. The accuracy of the measurement, measured in terms of mean relative error and standard deviation, underscores the reliability of our I-V tracer. The prototype exhibits a maximum mean error of approximately 3% in mono-Si technology, demonstrating a remarkable alignment between the measured and theoretical data.

One of the key distinguishing features of our I-V tracer lies in its innovative disconnecting circuit. Unlike commercial devices and most of the tracers proposed in the literature, our instrument allows for the continuous monitoring of PV modules within strings without the need to shut down power generation and string disassembly. Thanks to the wireless communication, our instrument can enable continuous data collection over extended periods without the need for frequent recalibration or maintenance. Nevertheless, the data collected can be conveniently accessed and analyzed through a user-friendly GUI, facilitating a comprehensive evaluation of the PV performance.

Future research efforts may focus on further enhancing the functionality, including integration with advanced data analytics algorithms for predictive maintenance and fault detection. With some modifications, this tool can also be used for large PV settings, such as string and arrays, by using the same circuit topology with minor hardware adjustments for higher voltage and power capacities.

## 5. Conclusions

This paper focuses on the design and development of a novel low-cost and battery-powered I-V tracer for the in situ monitoring of PV modules. The tool offers advanced functionalities, including the ability to trace the I-V curve of the tested module during its normal operation within the string, thanks to a specific disconnecting circuit. It incorporates a wireless and low-power communication module to trigger the measurements on-demand via a user-friendly GUI. The control unit of the device is based on a microcontroller, implementing an efficient control algorithm to evenly distribute the measurement points along the I-V curve. The realized prototype is tailored for testing a medium-power PV module with voltage and current capacities of 40 V and 10 A, respectively. Since the proposed device is based on a linear circuit topology, a potential limitation can arise in terms of thermal management, because the energy produced by the PV module under test is fully dissipated, partly by the resistive load and partly by the Darlington transistors. Consequently, in the case of PV modules of larger capacities, the device necessitates proper cooling systems, such as heatsinks, thus leading to an increase in the weight and overall cost. The effectiveness of the device is validated through an experimental campaign comprising a set of six PV modules of distinct technologies under different levels of irradiance and temperature. The experiments show the accuracy of our instrument in capturing the I-V curves both in uniform and partial shading conditions, with a maximum relative error of 3%.

## Figures and Tables

**Table 1:** Electrical parameters of the tested PV modules under standard test conditions.

	N-Type Mono-Si		Mono-Si		a-Si Thin Film	Poly-Si
	#1	#2	A	B		
P_rated_ [W_p_]	415		20	100	100	120
*V_OC_* [V]	37.67		21.6	22.5	23.75	22.0
*I_SC_* [A]	13.95		1.1	5.75	5.5	7.93
V_MPP_ [V]	31.81		18.5	18.9	19.8	17.5
I_MPP_ [A]	13.05		0.7	5.29	5	6.98
Efficiency [%]	21.3		19.2	19.2	23	17
T coefficient P_MAX_ [%/°C]	−0.30		−0.44	−0.44	−0.2	−0.5
Bifaciality factor	0.8		-	-	-	-

**Table 2:** Key components used in the developed I-V curve tracer.

		Description	Model
MCU		16-bit, 70 MHz clock speed	DSPIC33EP256GM604-I-PT
Current Sensor		Hall-sensor IC	LEM HY 15-P
Voltage Sensor		Resistive voltage divider	-
DAC		12-bit double-channel	MCP4822-E/MS
NPN Darlington	Leg 1	90 V, 50 A	MJ11032G
	Leg 2	100 V, 20 A	MJH6284G
PNP Darlington		60 V, 4 A	BD678
R_1_		1 Ω, 100 W	Ohmite TEH100M1R00FE
R_2_		10 Ω, 100 W	Ohmite TEH100M10R0FE
M_SC_		80 V, 120 A	PSMN2R8-80BS
M_DISC_		80 V, 120 A	PSMN2R8-80BS
D_BYP_		100 V, 30 A	VS-30CPQ100PBF
BT		-	LAIRD TECNOLOGIES BT740-SC
Battery		3.7 V, 2050 mAh, 7.59 Wh	-

**Table 3:** Economic assessment of the proposed I-V tracer against commercial devices.

	Price (EUR)
Proposed I-V tracer	355.00
Amprobe Solar-600	2048.05
RS ISM 490A	1261.63
Seaward PV200	1720.80
DS-100C	5298.22

**Table 4:** Outdoor experimental results of the different tested PV modules.

		G_f_ [W/m^2^]	G_b_ [W/m^2^]	T_AMB_ [°C]	T_PV_ [°C]	*V_OC_* [V]	*I_SC_* [A]	P_MAX_ [W]
N-type mono-Si	#1	710	120	26	38	36.35	9.76	283.5
		502	101	24	35	35.69	7.09	204.2
		303	80	21	28	36.01	4.18	121.76
	#2	709	119	26	38	36.01	8.13	208.2
		502	101	24	35	35.15	5.80	126
		320	82	21	28	35.60	3.48	60.2
Mono-Si	A	711	-	26	39	19.94	0.24	2.95
		502	-	24	38	19.42	0.17	1.65
		299		21	31	18.49	0.10	0.59
	B	711	-	26	39	20.65	3.81	44.79
		510	-	24	37	20.29	2.71	30.09
		312	-	21	31	19.94	1.65	20.59
a-Si thin film		699	-	26	38	22.62	3.82	62.25
		510	-	24	35	22.45	2.82	45.58
		305	-	21	28	22.17	1.63	27.75
Poly-Si		700	-	26	39	21.04	5.99	88.94
		510	-	24	35	20.67	4.32	63.05
		305	-	21	28	20.00	2.58	36.58

**Table 5:** Mean and standard deviation of the obtained results for the different tested PV modules.

	Bifacial N-Type Mono-Si		Mono-Si		a-Si Thin Film	Poly-Si
	#1	#2	A	B		
Mean [%]	0.94	1.69	2.13	3.04	1.28	0.38
Standard Deviation [%]	5.14 × 10^−15^	0.16	0.25	0.41	0.15	0.045

## Data Availability

Data are available on reasonable request to the corresponding author of the manuscript (M.D., mahmoud.dhimish@york.ac.uk).
